# Global functional connectivity density alterations in patients with bipolar disorder with auditory verbal hallucinations and modest short‐term effects of transcranial direct current stimulation augmentation treatment—Baseline and follow‐up study

**DOI:** 10.1002/brb3.1637

**Published:** 2020-04-18

**Authors:** Chuanjun Zhuo, Feng Ji, Xiaodong Lin, Hongjun Tian, Lina Wang, Yong Xu, Wenqiang Wang, Deguo Jiang

**Affiliations:** ^1^ School of Mental Health Jining Medical University Jining China; ^2^ Psychiatric‐Neuroimaging‐Genetics Laboratory Wenzhou Seventh People's Hospital Wenzhou China; ^3^ Psychiatric‐Neuroimaging‐Genetics‐Comorbidity Laboratory Tianjin Mental Health Centre Tianjin Anding Hospital Mental Health Teaching Hospital of Tianjin Medical University Tianjin China; ^4^ Department of Psychiatry First Hospital/First Clinical Medical College of Shanxi Medical University Taiyuan China; ^5^ MDT Center for Cognitive Impairment and Sleep Disorders First Hospital of Shanxi Medical University Taiyuan China; ^6^ Co‐collaboration Laboratory of China and Canada Xiamen Xianyue Hospital and University of Alberta Xiamen China

**Keywords:** auditory verbal hallucination, bipolar disorder, gFCD, hippocampus, tDCS

## Abstract

**Objectives:**

To investigate the neuroimaging characteristics of auditory verbal hallucinations (AVHs) in patients with bipolar disorder (BP) experiencing depressive episodes with and without AVHs, and alterations in those characteristics after transcranial direct current stimulation (tDCS).

**Methods:**

For a baseline pilot study, we recruited 80 patients with BP and depressive status (40 with and 40 without AVHs), and 40 healthy controls (HCs). Their global functional connectivity density (gFCD) was screened by functional magnetic resonance imaging (fMRI). Voxel‐wise one‐way analysis of covariance (ANCOVA) was conducted to detect intergroup differences in gFCD. In a follow‐up study, the effects of 5 weeks of tDCS augmentation treatment on clinical symptoms and gFCD were assessed in the 40 BP patients with AVHs.

**Results:**

Compared to HCs, BP patients with and without AVHs exhibited increased gFCD in the central parietal lobe, insular lobe, and middle cingulate cortex, with decreased gFCD in the posterior parietal cortex, lateral prefrontal cortex, and occipital lobe (all bilateral). Only patients with AVHs showed increased gFCD in the Broca and Wernicke regions, and decreased gFCD in the hippocampus (all bilateral). After 5 weeks of tDCS, AVHs were slightly alleviated and gFCD abnormalities in the hippocampus were mildly attenuated.

**Conclusions:**

Patients with BP and AVHs showed disturbances in the brain's communication capacity mainly in the left frontoparietal network, control network, and memory circuitry. Five weeks of tDCS alleviated AVHs slightly, without improving depressive symptoms, and attenuated hippocampal gFCD alterations in these patients.

## Graphical abstract

To the best of our knowledge, this report is the first to date to describe gFCD alterations in patients with BD with and without AVHs and to investigate the effects of tDCS augmentation treatment on AVHs in patients diagnosed with BD. Our findings suggest that there are information communication disturbances affecting the left frontoparietal network, control network, and memory circuitry in patients with BD and AVHs. Short‐term (5‐week) tDCS augmentation treatment alleviated AVHs modestly in this patient population while reversing BD‐associated gFCD alterations in the hippocampus. Going forward, it will be important to optimize tDCS treatment regimens for patients with BD.

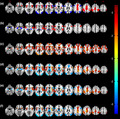

## 1 INTRODUCTION

Auditory verbal hallucinations (AVHs) are psychotic symptoms characterized by the auditory perception of speech in the absence of external auditory verbal stimuli (de Leede‐Smith & Barkus, 2013). They can occur in patients with psychotic disorders, including schizophrenia (SCZ) and bipolar disorder (BP), among others, as well as in healthy individuals (Upthegrove et al., 2016). Some 11.3%–62.8% of patients with BP report experiencing AVHs (Toh, Thomas, & Rossell, 2015). AVHs can worsen BP symptoms and lead to high‐risk behaviors, including self‐harm and suicide. Thus, elucidation of the neural characteristics of AVHs in the context of BP can help to improve treatment planning for, and thus prognosis of, BP.

Most people who experience AVHs have detectable brain connectivity network alterations observable in functional imaging studies (Curcic‐Blake et al., 2017). A meta‐analysis showed that AVHs were associated with activity changes in circuitry linking the precentral gyrus, insula, parietal lobe, and hippocampus (Jardri, Pouchet, Pins, & Thomas, 2011). Conversely, AVHs have been associated with dysconnectivity among the anterior cingulate cortex, insular cortex, and other cortical regions (Chang et al., 2017). Additionally, AVH‐related connectivity disturbances have been observed in the posterior temporal, inferior frontal, and parietal lobes (Catani et al., 2011), as well as between the claustrum and insula in a resting‐state (Mallikarjun et al., 2018). During AVH episodes, cortex‐subcortex connectivity alterations have been observed and fluorodeoxyglucose positron emission tomography (PET) has revealed abnormal resting‐state auditory cortex‐thalamus connectivity (Horga et al., 2014). Because most of the aforementioned studies focused on functional connectivity strength in patients on the SCZ spectrum, however, they may not generalize to patients with other disorders (Hugdahl & Sommer, 2018; Mallikarjun et al., 2018; Scheinost, Tokoglu, Hampson, Hoffman, & Constable, 2019; Steinmann, Leicht, & Mulert, 2019; Tomasi & Volkow, 2010; Zhao et al., 2018). Consistent findings of functional disturbances affecting Wernicke's and Broca's areas in patients with AVHs support the notion that speech and language processing areas are likely to be involved in the experience of AVHs (Zmigrod, Garrison, Carr, & Simons, 2016). However, it is unclear whether the lateralization of AVHs seen in SCZ (Sommer, Aleman, & Kahn, 2003) would be present outside the context of SCZ. There have been substantial investigations of the brain features of AVHs (Di Biase et al., 2020; Kubera et al., 2019; Steinmann et al., 2019) and AVH treatment strategies (Craig et al., 2018; Javitt & Sweet, 2015; Kantrowitz et al., 2019; Kubera, Barth, Hirjak, Thomann, & Wolf, 2015; Thomas, Moulier, Valero‐Cabre, & Januel, 2016; Thomas et al., 2019; Zhuo, Xu, Zhang, Jing, & Zhou, 2019) in patients with SCZ, whereas such investigations have been lacking in the BP patient population. Most of the data collected to date regarding AVHs in patients with BP are limited to prevalence (Toh et al., 2015), clinical features of AVHs (Smith, Johns, & Mitchell, 2017), predictors of AVH persistence (Jenkins, Bodapati, Sharma, & Rosen, 2018), and the relationship between AVHs and mood and/or cognitive ability (David & Lucas, 1993; Jenkins et al., 2018; Rass et al., 2010; Smith et al., 2017; Toh, Castle, Thomas, Badcock, & Rossell, 2016).

Transcranial direct current stimulation (tDCS) has been reported to produce dramatic improvements in AVHs in patients with SCZ (Bose et al., 2018; Brunelin et al., 2012; Chang, Tzeng, Chao, Yeh, & Chang, 2018; Mondino et al., 2016; Ponde et al., 2017), to improve cognitive functions in patients with BP (McClintock et al., 2020), and to improve depressive symptoms in patients with BP (Mutz, Edgcumbe, Brunoni, & Fu, 2018). Some neuroimaging studies have demonstrated tDCS‐induced alterations in brain activity that were associated with improvement of BP symptoms (Bertocci et al., 2019; Tortella et al., 2015). Altogether, such findings suggest that tDCS augmentation treatment may be an optimal treatment modality for patients with BP who experience AVHs. Given the high prevalence of AVHs among patients with BP, characterization of AVH‐specific aberrant functional connectivity in subjects with BP documentation of tDCS augmentation treatment effects on AVHs in BP can improve our understanding of the neural mechanisms of AVHs and provide information that can be useful for optimizing treatment strategies for patients with BP and AVHs.

Global functional connectivity density (gFCD), which represents the number of connections between a single voxel and other voxels throughout the brain, can be used as a quantitative index of local neural activity (Lang et al., 2015). Moreover, given that PET findings indicate that gFCD is a potential biomarker of quantitative changes in glucose metabolism (Thompson et al., 2016), gFCD alterations may reflect both broad information communication capability throughout the brain while also being useful as a qualitative index of local metabolism within brain regions (Qin, Xuan, Liu, Jiang, & Yu, 2015; Zhang et al., 2016; Zhuo et al., 2014, 2017). For example, in studies of patients with SCZ, Huang et al. reported that gFCD was increased within the default mode network following electroconvulsive therapy (Huang et al., 2018) and Chen et al. reported functional connectivity density alterations between the visual cortex and sensorimotor cortex (Chen et al., 2015).

Here, we report a two‐part study encompassing a baseline pilot study and a follow‐up study exploring aberrant gFCD patterns in patients with BP who experience depressive episodes with AVHs (Craig et al., 2018; Di Biase et al., 2020; Javitt & Sweet, 2015; Kantrowitz et al., 2019; Kubera et al., 2015, 2019; Steinmann et al., 2019; Thomas et al., 2016, 2019; Zhuo et al., 2019). In the baseline study, we conducted a quantitative analysis of brain functional connectivity features and metabolic alterations characteristic of BP‐related AVHs. In the follow‐up study, we examined how tDCS augmentation treatment affected AVH symptoms in the context of BP and whether there are corresponding brain functional alterations induced by tDCS (Bertocci et al., 2019; Bose et al., 2018; Brunelin et al., 2012; Chang et al., 2018; Craig et al., 2018; Kantrowitz et al., 2019; McClintock et al., 2020; Mondino et al., 2016; Mutz et al., 2018; Ponde et al., 2017; Tortella et al., 2015). In light of prior related findings, we tested the following three hypotheses: (1) patients with BP experiencing depressive episodes and AVHs will have gFCD alterations in brain regions involved in the language, memory, and mood regulation; (2) tDCS augmentation treatment can alleviate AVH symptoms; and (3) tDCS augmentation treatment can normalize gFCD aberrations in this patient population.

## 2 MATERIALS AND METHODS

### 2.1 Subjects

Because patients in hypomanic and manic states may have poor compliance with neuroimaging, we examined patients with BP in a depressive state. We recruited 80 such patients (40 with and 40 without AVHs during depressive episodes) and 40 healthy controls (HCs) from Wenzhou Seventh People's Hospital between 1 June 2014 and 31 December 2018. All study participants were of Han ethnicity and right‐handed. Two senior psychiatrists diagnosed BP using the Structured Clinical Interview of the DSM‐IV (SCID‐IV) (First, First, Spitzer, & Gibbon, 2002) and screened HCs with the non‐patient edition (SCID‐I‐NP) (B., Gibbon, Spitzer, & Williams, 1997) and the Structured Clinical Interview for DSM‐IV Axis II Personality Disorders (SCID‐II) (First & Gibbon, 1997); HCs were also assessed to confirm that they had no first‐degree relatives with a history of mood‐ or SCZ‐related disorders with the Family Interview for Genetic Studies (FIGS) (NIMH Genetics Initiative: Family Interview for Genetic Studies (FIGS), 1992), a clinician‐administered symptom checklist screening tool that probes family members’ diagnoses. The patient enrollment criteria were diagnosis of BP with a current depressive episode and diagnosis of AVHs was performed according to Ratcliff et al.’s recommendations using the auditory hallucination rating scale (AHRS), which identifies AVHs and assesses AVH symptom severity (Haddock, McCarron, Tarrier, & Faragher, 1999; Ratcliff, Farhall, & Shawyer, 2011). The exclusion criteria for BP patients were: current mania/hypomania symptoms as indicated by a Young Mania Rating Scale (YMRS) (Young, Biggs, Ziegler, & Meyer, 1978) score ≤ 5; neurological or physical disease that can affect brain functional activity, including endocrine diseases, other mental disorders (e.g., schizoaffective disorder, major depressive disorder, etc.), and substance abuse; any scanning contraindication (including claustrophobia), history of unconsciousness for more than 5 min from any cause, and left handedness determined by the Annett Hand Preference Questionnaire (Tapley & Bryden, 1985). Written informed consent was obtained from each participant or his or her legal guardian. The Ethics Committee of Wenzhou Seventh People's Hospital approved this study (No. ZS2017011).

### 2.2 Assessment of BP and AVH symptom severity

The severity of depressive symptoms was evaluated with the Hamilton Depression Rating Scale (HAMD, version 17) (Faravelli, Albanesi, & Poli, 1986), which is designed for adults and used to evaluate the severity of depressed mood, feelings of guilt, suicidal thoughts, insomnia, agitation, anxiety, weight loss, and somatic symptoms. Each item was scored on a 3‐ or 5‐point scale, and completion of the questionnaire takes about 20 min. Manic and hypomanic symptoms were assessed by YMRS (Young et al., 1978). AVH symptom severity was evaluated with the AHRS (Haddock et al., 1999).

### 2.3 Neuroimaging

Functional magnetic resonance imaging (fMRI) was performed with a GE Healthcare Discovery MR750 3‐T MRI system (General Electric, Milwaukee, WI) with an eight‐channel phased‐array head coil. The participants were instructed to lie in a supine position and to minimize thinking and head movements during scanning. The neuroimaging parameters were as follows: 2,000‐ms repetition time (TR), 45‐ms echo time (TE), 32 slices, 4‐mm slice thickness, 0.5‐mm gap, 220 × 220 field of view, 64 × 64 acquisition matrix, and 90° flip angle. The scans were acquired with parallel imaging and SENSitivity Encoding (factor = 2). Structural images were obtained with a high‐resolution three‐dimensional turbo‐fast echo T1‐weighted sequence with the following parameters: 8.2/3.2‐ms TR/TE, 170 slices, 1‐mm thickness, no gap, 256 × 256 field of view, 256 × 256 acquisition matrix, and 12° flip angle.

### 2.4 fMRI data preprocessing

Resting‐state fMRI data preprocessing was completed in SPM8 software (http://www.fil.ion.ucl.ac.uk/spm). For each subject, we did not include the first 10 volumes collected to allow time for signal equilibration and for subjects to adapt sensorially to scanning. Subsequent volumes were corrected for the inter‐slice acquisition delay and realigned to correct for subject movement. None of the subject's fMRI data exceeded predefined translational (<2 mm) or rotational (<2°) motion parameters. Fraction displacement (an index of volume‐to‐volume changes in head position between patients) did not differ significantly (t = 0.57*, p* = .56) between BP patients (0.120 ± 0.005) and HCs (0.109 ± 0.005). Several nuisance covariates (six motion parameters, their first derivations, and mean ventricular and white matter signals) were regressed out. Because a signal spike due to head motion can still disrupt fMRI results even after regressing out linear motion parameters (First & Gibbon, 1997), we further regressed out spike volumes for volumes with fraction displacements > 0.5. Following dataset band‐pass filtering (frequency range, 0.01–0.08 Hz), we undertook normalization, wherein structural images were co‐registered linearly first with the mean functional image and then with Montreal Neurological Institute (MNI) space. Each filtered volume was spatially normalized to MNI space based on its associated co‐registration parameters and then resampled into a 3‐mm cubic voxel.

### 2.5 gFCD

An in‐house Linux script was employed to calculate a gFCD value for each voxel^60^. Functional connectivity was compared between voxels with Pearson's linear correlation analysis (correlation coefficient threshold, *R* > 0.6). gFCD calculations were based on the whole‐brain gray matter volume, and a growth algorithm was used to calculate the gFCD for each voxel (x0), based on the total number of functional connections [k(x0)] between x0 and all other voxels. Each gFCD value was divided by the mean gFCD of all included voxels to increase the normality of the gFCD value distribution. FCD maps were smoothed with a 6 × 6 × 6‐mm^3^ Gaussian kernel to minimize interbrain differences in functional anatomy.

### 2.6 Follow‐up study procedures

Inspired by reported effects of tDCS on AVH in patients with SCZ (Chang et al., 2018; Mondino et al., 2016), we administrated tDCS augmentation treatment to patients with BP and AVH. They received a mood stabilizer and antidepressant agent daily combined with 5 weeks of tDCS augmentation treatment. The tDCS was administered with an Eldith DC stimulator (Neuroconn DC Stimulator Plus) from GmbH (Ilmenau, Germany) with two 7 × 5‐cm sponge‐type electrodes saturated with 0.9% saline solution. Consistent with previous studies (Bertocci et al., 2019; Bose et al., 2018; Brunelin et al., 2012; Chang et al., 2018; Kantrowitz et al., 2019; McClintock et al., 2020; Mondino et al., 2016; Mutz et al., 2018; Ponde et al., 2017; Tortella et al., 2015) and based on the international 10‐to‐20 electrode placement scheme, we placed the anode center midway between the F3 and FP1 electrode locations, corresponding to the left prefrontal/dorsolateral prefrontal area. The cathode was situated midway between T3 and P3, over the left temporoparietal junction area. Two‐milliampere stimulation was applied for 20 min, twice daily on five consecutive days (Monday through Friday) for the 5‐week treatment period. In sham stimulation, the current was turned on for 30 s and then ramped down to 0 mA where it remained for the rest of the 20‐min treatment period.

### 2.7 Statistical analysis

Participants’ sociodemographic data were analyzed with one‐way analyses of variance. Depressive symptom severity and duration of illness were compared between patient groups with *t* tests. Group differences in gFCD were detected with voxel‐wise one‐way analyses of covariance (ANCOVAs), with age, sex, and education level as covariates, followed by post hoc intergroup comparisons. Family‐wise error (FWE) correction for multiple comparisons was performed with a significance threshold of *p* < .05.

To investigate the relationship between gFCD values and total AHRS scores, a voxel‐wise multiple regression analysis was conducted; regions showing significant gFCD differences in the BP with AVH group were compared with the same regions in the other groups. Sex, age, and education level were considered to be nuisance covariates. We also examined the correlation between gFCD and AHRS score within the 40 patients with BP and AVH specifically, with FWE correction for multiple comparisons. All statistical tests were performed in SPSS (v. 22.0, IBM Corp, Armonk, NY) with a significance level of *p* < .05.

## 3 RESULTS

### 3.1 Demographic and clinical characteristics of the subjects

Sociodemographic information for the three study groups, namely BP patients with AVH (BP‐AVH), BP patients without AVH (BP‐noAVH), and HCs, is provided in Table 1. The three groups did not differ in terms of gender, age, or education level (Table 1). The BP‐AVH and BP‐noAVH groups did not differ in terms of the severity of their depressive symptoms or illness duration (*p* = .272).

**TABLE 1:** Demographic and clinical characteristics of the subjects by group

Characteristic	BP‐AVH (depressive state) (*N* = 40)	BP‐noAVH (depressive state) (*N* = 40)	HCs (*N* = 40)	*p*
Age, years	40.5 ± 11.0	42.5 ± 15.5	42.0 ± 12.5	.711
Gender, females/males	15/25	13/27	16/24	.625
Educational level years	13.5 (2.6)	14.2 (3.2)	15.0 (1.4)	.121
Illness duration, months	40.5 ± 10.5	42.0 ± 12.2	–	.272
HAMD score	36.5 ± 5.5	32.0 ± 7.5	–	.299
Duration of current depressive episode, weeks	7.7 ± 1.5	7.5 ± 2.0	–	.337
YMRS score	2.8 ± 1.2	2.7 ± 1.4	–	.677
AHRS score	18.7 ± 5.5	–	–	–

### 3.2 gFCD differences among groups

**FIGURE 1:**
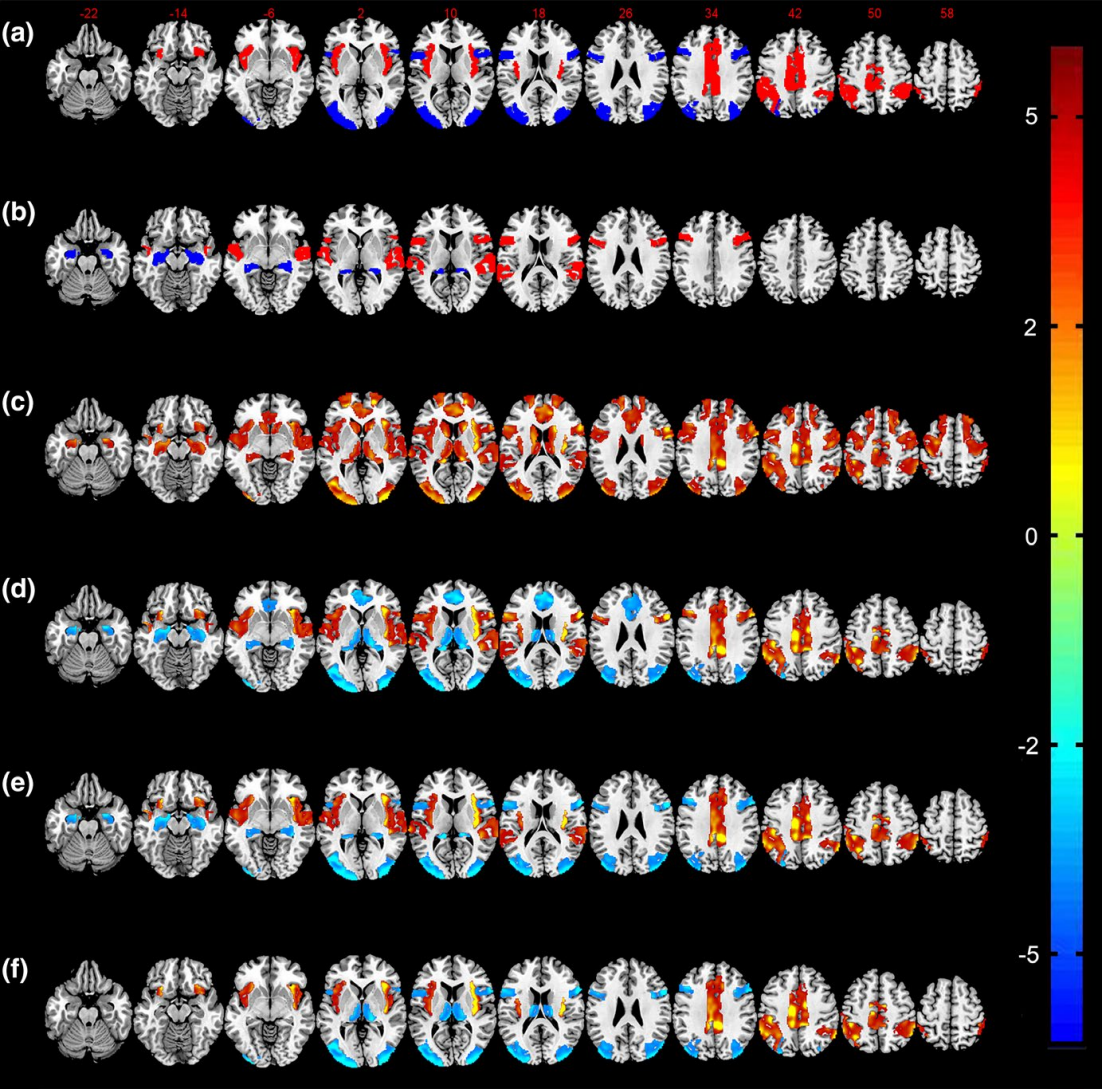
gFCD differences among BP‐AVH, BP‐noAVH, and HCs groups. (a) Differences in gFCD between the two BP patient groups and HCs. (b) Differences in gFCD between BP‐noAVH and BP‐AVH groups. (c) Differences in gFCD between BP‐AVH group and HCs. (d) Differences in gFCD between BP‐noAVH group and HCs. (e) Common aberrant gFCD pattern for BP with and without AVH. (f) Distinct aberrant gFCD pattern for BP with AVH

Voxel‐wise ANCOVAs revealed intergroup differences in gFCD in the precentral gyrus, postcentral gyrus, temporal lobe, insular lobe, middle cingulate gyrus, occipital lobe, anterior cingulate cortex, hippocampus, thalamus, and caudate nucleus (all bilateral; all FWE‐corrected *p*＜0.05; Figure 1a). Compared with the BP‐noAVH group, the BP‐AVH group demonstrated significantly increased gFCD in the central parietal lobe, insular lobe, Broca region, Wernicke region, and middle cingulate gyrus, as well as decreased gFCD in the posterior parietal cortex, medial prefrontal cortex, occipital lobe, thalamus, and hippocampus (all bilateral; Figure 1b). Compared with HCs, patients in BP‐AVH group had significantly increased gFCD values in the central parietal lobe, insular lobe, temporal lobe, and middle cingulate cortex, together with decreased gFCD values in the posterior parietal cortex, lateral prefrontal cortex, occipital lobe, and hippocampus (all bilateral; Figure 1c). Finally, compared with HCs, patients in the BP‐noAVH group had significantly increased gFCD values in the central parietal lobe, insular lobe, and middle cingulate cortex, together with decreased gFCD values in the posterior parietal lobe, lateral prefrontal cortex, occipital lobe, and thalamus (all bilateral; Figure 1d).

Relative to HCs, patients in both BP groups had increased gFCD in the postcentral gyrus, insular lobe, and middle cingulate cortex, and decreased gFCD in the posterior parietal cortex, lateral prefrontal cortex, and occipital lobe (all bilateral). We defined these alterations as the common aberrant gFCD pattern for BP with and without AVH (Figure 1e). Compared with the other two groups, patients in the BP‐AVH group demonstrated increased gFCD values in the Broca and Wernicke regions, and decreased gFCD in the hippocampus (all bilateral). These alterations thus formed a distinct aberrant gFCD pattern for BP with AVH (Figure 1f).

### 3.3 Association of gFCD with AVH severity

No significant correlation between gFCD and AVH severity (ARHS total score and frequency) was observed in the BP‐AVH group.

### 3.4 Follow‐up after tDCS

**FIGURE 2:**
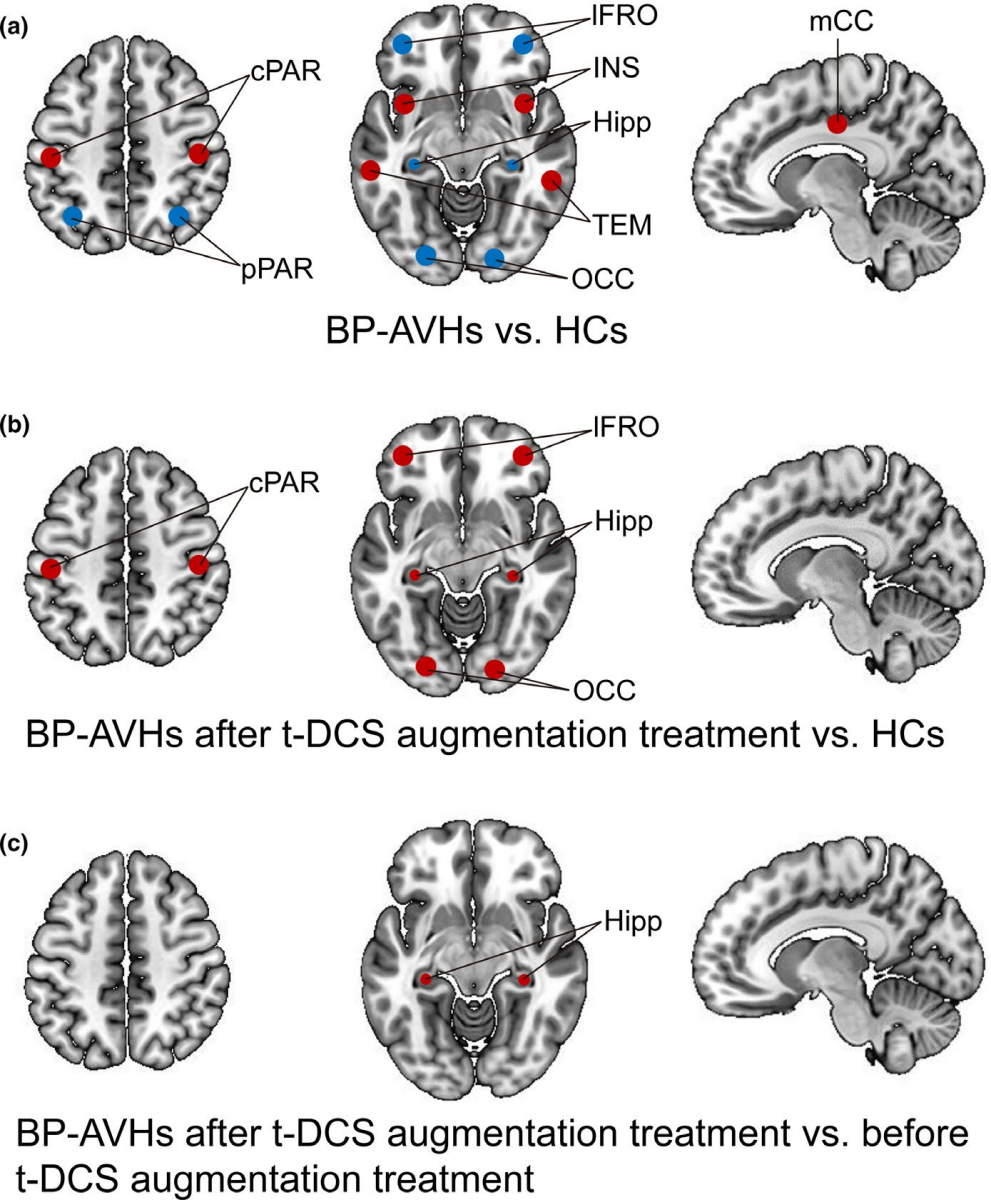
Post‐tDCS treatment associated changes in gFCD in the BP‐AVH patient group (*N* = 40)

After 5 weeks of tDCS augmentation treatment, we observed a significant 23.5% reduction in AVH symptom severity in BP‐AVH patients (Table 2), without a significant change in depressive symptoms (Table 2). Meanwhile, marked changes in gFCD were observed in this patient group following tDCS augmentation therapy, including gFCD decreases in the postcentral gyrus, lateral prefrontal cortex, occipital lobe, and hippocampus with matched HCs as a reference group (Figure 2). Compared to pretreatment baseline data, after 5 weeks of tDCS treatment, only hippocampal gFCD had increased significantly in the BP‐AVH patients. None of the patients in the follow‐up study experienced any major adverse events.

**TABLE 2:** Clinical effects of tDCS augmentation treatment in BP and AVH groups

Characteristic	Before treatment *N* = 40	After treatment *N* = 40	*t*	*p*
AHRS score	18.7 ± 5.5	14.3 ± 3.7	1.566	.039
HAMD score	36.5 ± 5.5	34.9 ± 8.6	0.598	.350
YMRS score	2.8 ± 1.2	2.9 ± 0.5	0.256	.698

## 4 DISCUSSION

The present results revealed a common gFCD pattern for BP in general (with and without AVH) as well as a distinct gFCD pattern for BP patients with AVH. The distinct aberrant pattern for BP with AVH involved primarily components of language and memory processing circuits (Figure 1f, Table 3). After 5 weeks of tDCS augmentation treatment, we observed a modest alleviation in AVH severity. More notably, following the tDCS treatment, only BP patients with AVH exhibited an increase in gFCD in the hippocampus relative to baseline data (self‐comparisons).

**TABLE 3:** Cluster size, cluster coordinates, and T values of distinct gFCD aberrations for BP patients with AVH

Region	Cluster size	Cluster coordinates			*T*
		*x*	*y*	*z*	
Broca region (left)	26	44	18	9	3.813
Broca region (right)	24	−45	18	8	3.845
Wernicke region (left)	28	25	−6	2	3.920
Wernicke region (right)	25	22	−6	2	3.895
Hippocampus (left)	30	−25	−14	10	−3.790
Hippocampus (right)	32	24	−14	10	−3.331

The presently observed, bilaterally reduced gFCD in the hippocampus in BP‐AVH patients supports the unstable memory hypothesis, which holds that a failure to control the contents of memories may cause remembered experiences to emerge into consciousness (Curcic‐Blake et al., 2017). Aberrant hypoactivity of the hippocampus, a key mediator of memory processes, can thus mediate memory control deficits, which may be the basis of so‐called unstable memories. Moreover, our follow‐up study finding showing an apparent amelioration of the BP‐AVH patients’ gFCD deficit after tDCS augmentation treatment provides further new evidence in support of the unstable memory hypothesis. It has been suggested that disturbances in the reciprocal interactions between memory and language processing areas may constitute the basis of AVH (Curcic‐Blake et al., 2017). Accordingly, hyperactivity in the Wernicke and Broca areas, which are major hubs of language processing, can disturb this processing and, potentially, thus lead to AVH (Price, 2010). Our findings are also consistent with the source monitoring hypothesis, which proposes that abnormal memory‐related activity leads to failures in stimulus‐feature binding and memory retrieval, disrupting one's ability to form a cohesive representation of an experience, which has been suggested to underlie the development of AVH (Mitchell & Johnson, 2009). Finally, our findings of broad increases in gFCD in many brain regions in BP‐AVH patients also support the interhemispheric miscommunication hypothesis, which holds that increased interhemispheric synchrony between auditory areas may contribute to AVH development (Eggermont, 2007).

The common aberrant gFCD pattern for BP identified in the present study is largely consistent with previous findings regarding functional connectivity alterations in BP (Doucet, Bassett, Yao, Glahn, & Frangou, 2017; He et al., 2017; Vai, Bertocchi, & Benedetti, 2019), and the regions involved are similar to regions that have been implicated in BP previously (Baethge et al., 2005; He et al., 2017; Nguyen et al., 2017; Smith et al., 2017; Vai et al., 2019; Wang et al., 2019; Waters, Woods, & Fernyhough, 2013). Thus, our common aberrant gFCD pattern can be taken to represent a set of neuroimaging features associated with BP (Baethge et al., 2005; Nguyen et al., 2017; Smith et al., 2017; Wang et al., 2019; Waters et al., 2013). Moreover, our findings of a lack of improvement in depressive symptoms following tDCS together with a persistence of gFCD alterations in these common aberrant regions provide further indirect evidence that the BP pattern observed here (bilateral gFCD increases in the postcentral gyrus, insular lobe, and middle cingulate cortex, with decreases in the posterior parietal cortex, lateral prefrontal cortex, and occipital lobe) may constitute functional features of BP.

### 4.1 Limitations

There are several limitations of this two‐part study that must be considered when interpreting the results. First, the validity of our follow‐up study methods should be confirmed directly and empirically in additional research. Second, we employed a relatively short‐duration intervention (5 weeks), which may not be enough to achieve full clinical and gFCD benefits. We will pursue longer term (>3 months) studies to further clarify the effects of sustained tDCS augmentation therapy. Third, for the purposes of the present study, we included only BP patients with AVH in the follow‐up study, which generates an inherent sample bias. Treatment effects in BP patients both with and without AVHs should be examined in larger studies. Fourth, the small sample size of each group limits the significance of the findings. Fifth, our selection of only subjects with BP experiencing depressive episodes to improve neuroimaging compliance (Carta, Paribello, Nardi, & Preti, 2019) may introduce a sample bias. In future studies, we plan to enroll hypomanic patients with BP and AVH to enable fuller characterization of the features identified in the present study. Sixth, we could not account for the effects of therapeutic medications on gFCD. Patients with BP have complex therapeutic medication regimes, including various mood stabilizers, antidepressants, antipsychotics, and antianxiety drugs; no equivalence equation is available to enable normalization to a single antipsychotic agent. Hence, as suggested by Flavie et al. and Upthegrove et al., we are also performing studies in drug‐naïve patients to identify drug‐related factors in this patient population (Upthegrove et al., 2016; Waters & Fernyhough, 2017).

## 5 CONCLUSIONS

To the best of our knowledge, this report is the first to date to describe gFCD alterations in patients with BP with and without AVH and to investigate the effects of tDCS augmentation treatment on AVH in patients diagnosed with BP. Our findings suggest that there are information communication disturbances affecting the left frontoparietal network, control network, and memory circuitry in patients with BP and AVH. Short‐term (5‐week) tDCS augmentation treatment alleviated AVH modestly in this patient population while reversing BP‐associated gFCD alterations in the hippocampus. Going forward, it will be important to optimize tDCS treatment regimens for patients with BP.

## CONFLICT OF INTEREST

The authors declare that they have no conflict of interest.

## AUTHOR CONTRIBUTIONS

CZ, FJ, YL, and DJ conceived and designed research; XL, HT, and LW collected data and conducted research; YX, WW, and DJ analyzed and interpreted data; HT and WW wrote the initial paper; CZ and YX revised the paper; DJ had primary responsibility for final content. All authors read and approved the final manuscript.

## Data Availability

The datasets generated and analyzed during the present study are available from the corresponding author on reasonable request.
